# NADPH oxidase 5 is a novel susceptibility gene for type 2 diabetes mellitus

**DOI:** 10.20945/2359-4292-2023-0527

**Published:** 2024-10-17

**Authors:** Iuliia Azarova, Elena Klyosova, Valentina Azarova, Alexey Polonikov

**Affiliations:** 1 Department of Biological Chemistry Kursk State Medical University Kursk Russian Federation Department of Biological Chemistry, Kursk State Medical University, Kursk, Russian Federation; 2 Laboratory of Biochemical Genetics and Metabolomics Research Institute for Genetic and Molecular Epidemiology Kursk State Medical University Kursk Russian Federation Laboratory of Biochemical Genetics and Metabolomics, Research Institute for Genetic and Molecular Epidemiology, Kursk State Medical University, Kursk, Russian Federation; 3 Department of Biology Medical Genetics and Ecology Kursk State Medical University Kursk Russian Federation Department of Biology, Medical Genetics and Ecology, Kursk State Medical University, Kursk, Russian Federation; 4 Kursk Emergency Hospital Kursk Russian Federation Kursk Emergency Hospital, Kursk, Russian Federation; 5 Laboratory of Statistical Genetics and Bioinformatics Research Institute for Genetic and Molecular Epidemiology Kursk State Medical University Kursk Russian Federation Laboratory of Statistical Genetics and Bioinformatics, Research Institute for Genetic and Molecular Epidemiology, Kursk State Medical University, Kursk, Russian Federation

**Keywords:** Type 2 diabetes, oxidative stress, NADPH oxidase 5, single nucleotide polymorphism, risk factors, gene-environment interactions

## Abstract

**Objective:**

This pilot study investigated whether single nucleotide polymorphisms (SNP) in the NOX5 gene (NADPH oxidase 5) are associated with the type 2 diabetes (T2D) risk.

**Subjects and methods:**

A total of 1579 patients with T2D and 1627 age- and sex-matched healthy subjects were recruited for this study. Genotyping of common SNPs, namely rs35672233, rs3743093, rs2036343, rs311886, and rs438866, was performed using the MassArray-4 system.

**Results:**

SNP rs35672233 was associated with an increased risk of T2D (OR = 1.67, 95% CI 1.29-2.17, FDR = 0.003). The H3 haplotype (rs35672233T-rs3743093G-rs2036343A-rs311886C-rs438866C) increased T2D risk (OR = 1.65, 95% CI 1.27-2.13, FDR = 0.001). The rs35672233 polymorphism and H3 haplotype were found to have an association with T2D risk only in subjects with a body mass index greater than 25 kg/m^2^ (FDR < 0.01). Environmental risk factors, such as chronic psycho-emotional stress, sedentary lifestyle, high-calorie diet and SNP rs35672233 were jointly associated with T2D susceptibility. A haplotype comprising the allele rs35672233-C and conferring protection against T2D, was associated with elevated levels of antioxidants such as total glutathione and uric acid, as well as reduced levels of two-hour postprandial glucose in the plasma of patients. The NOX5 polymorphisms showed no associations with diabetic complications.

**Conclusion:**

The present study is the first to establish associations between polymorphisms in NOX5 and the risk of type 2 diabetes mellitus, and provides a new line of evidence for the crucial role of oxidative stress-related genes in disease susceptibility.

## INTRODUCTION

Diabetes is a serious health problem that has grown in alarming magnitude and affects more than 500 million individuals worldwide ( 1 ). The primary molecular mechanisms underlying type 2 diabetes (T2D), the most widespread form of diabetes, are not well understood despite extensive research efforts over the last decades ( 2 , 3 ).

Oxidative stress is thought to be one of the main reasons for the onset of T2D, insulin resistance and associated complications ( 4 , 5 ). Free radicals or reactive oxygen species (ROS), biologically active molecules produced by immune cells and/or metabolic pathways, play a role in a variety of biological processes, such as cell-cell communication, defense against pathogen invasion, cell proliferation, autophagy, apoptosis, and aging ( 6 ). However, uncontrolled production of ROS is responsible for the development of oxidative stress, which triggers a chain reaction of harmful pathways leading to pancreatic beta cell dysfunction, insulin resistance, and diabetes ( 4 , 7 ). Identifying the genetic determinants that contribute to the disruption of redox homeostasis in type 2 diabetes mellitus will provide deeper insights into disease etiology and identify new targets for therapy and prevention.

The mitochondrial respiratory chain and NADPH (nicotinamide adenine dinucleotide phosphate) oxidase (NOX) enzymes are the primary sources of ROS production in cells including pancreatic beta cells ( 8 ). Single nucleotide polymorphisms (SNP) in genes encoding various subunits and isoforms of NOXs have been investigated as genetic markers of T2D ( 9 , 10 ) and its complications ( 11 , 12 ); however, the *NOX5* gene has not yet been the target of genetic association studies of diabetes. Increased expression of *NOX5* , leading to enhanced ROS production, was found to be associated with an increased risk of diabetic nephropathy ( 13 ) and vascular disease ( 14 ). The purpose of our pilot study was to investigate associations of *NOX5* polymorphisms with type 2 diabetes, biochemical parameters of redox homeostasis and glucose metabolism as well as explore the joint effects of the polymorphisms and known disease risk factors on T2D susceptibility.

## SUBJECTS AND METHODS STUDY PARTICIPANTS AND DIAGNOSIS OF TYPE 2 DIABETES

The Regional Ethics Review Committee of the Kursk State Medical University approved the study protocol (No.10, date: 12.12.2016), which complied with the ethical standards of the Declaration of Helsinki. Before enrollment, each subject provided written informed consent. A total of 3206 unrelated Russians were enrolled in the study, comprising 1627 age- and sex-matched healthy individuals (control group) and 1579 T2D patients. From November 2016 to October 2019, T2D patients were admitted to the Endocrinology Division of the Kursk City Clinical Emergency Hospital. The demographic, clinical, and laboratory features of the study groups are reported in our most recent paper ( 12 ). WHO criteria were used for the diagnosis of T2D: fasting blood glucose (FBG) level 7.0 mmol/L, random blood glucose level 11.1 mmol/L, and/or glycated hemoglobin (HbA 1 c) level 6.5% ( 15 ). The inclusion criteria for the patient group were as follows: 1) physician-verified disease supported by clinical, laboratory, and instrumental tests; and 2) age greater than 35 years. Patients were excluded from the study based on the following criteria: age < 35 years; lack of written informed consent to participate in the study; and three clinical conditions, including severe decompensation of T2D or coma, immune-mediated or idiopathic type 1 diabetes, gestational diabetes, MODY type of diabetes, and diseases of the exocrine pancreas, such as pancreatitis, trauma to the pancreas or pancreatectomy, pancreatic tumors, and hereditary diseases. The control group was recruited in our earlier studies and comprises healthy volunteers who arrived at the Kursk Blood Transfusion Station ( 16 , 17 ). The inclusion criteria for the control group were as follows: 1) absence of chronic diseases, 2) the 75-g oral glucose tolerance test and normal fast blood glucose levels, and 3) age > 35 years. A validated questionnaire ( 18 ) was used to conduct a personal interview survey with participants regarding patients’ social and family status; a thorough history of the illness and complaints; the age of disease onset; the number of episodes of disease decompensation; family history of diabetes; unhealthy habits (smoking and alcohol abuse); physical activity; stress from daily life; and the consumption of sweet, fatty, and high-calorie foods, proteins, fruits, and vegetables. Patients’ answers were classified as either appropriate or unfit for a healthy lifestyle according to WHO and ADA guidelines.

### Genetic analysis

Five milliliters of whole blood were drawn from each patient. Genomic DNA was extracted from whole blood samples using the robotic workstation QiaCube and QIAamp Blood Mini Kit (QIAGEN). Five SNPs of the *NOX5* gene, namely, rs35672233, rs3743093, rs2036343, rs311886, and rs438866, were selected for the study using the GenePipe tool ( https://snpinfo.niehs.nih.gov/snpinfo/selegene.html ) based on the functional characteristics of SNPs and haplotype structure of the gene in the European population from the HapMap project. The MALDI-TOF MassARRAY 4 system (Agena Bioscience Inc., San Diego, CA, USA) was used for SNP genotyping. The primer sequences are showed in Supplementary Table 1 . Genotyping was carried out without knowledge of the case-control status to ensure quality control. For repeat genotyping, 10% of the samples were randomly chosen and the repeatability test revealed a 100% concordance rate.

### Biochemical investigations

Additionally, fasting venous blood samples from a subgroup of patients were analyzes for the levels of plasma glutathione (258 T 2 D patients and 137 controls) and hydrogen peroxide (489 T 2 D patients and 153 controls). Aliquoted plasma samples were stored at -80°C until needed. Plasma was promptly deproteinized with trichloroacetic acid to determine glutathione levels. GSH/GSSG Ratio Detection test kit II (Cell Biolabs, USA; Abcam, USA) was used to assess glutathione levels using a fluorometric test procedure. The OxiSelectTM In Vitro ROS/RNS Assay Kit (Cell Biolabs, USA) was used to perform a fluorometric assay to measure the levels of hydrogen peroxide. The absorbance at 405 nm and fluorescence at 480 nm excitation/530 nm emission were measured using a Varioscan Flash microplate scanner (Thermo Fisher Scientific, USA).

### Statistical analysis

A genetic association study (GAS) power calculator ( http://csg.sph.umich.edu/abecasis/gas_power_calculator/ ) was used to assess statistical power for genetic association. Based on the sample sizes of 1579 individuals with T2D and 1627 healthy controls, an association analysis of the chosen polymorphisms with the risk of T2D might identify the genotype relative risk of 1.26-1.51 assuming 85-90% power and a 5% type I error (P = 0.05). The chi-square test was used to evaluate allele and genotype frequencies between patients and controls. Multiple logistic regression analysis was used to adjust for age, sex, and body mass index (BMI). Odds ratios (OR) and 95% confidence intervals (CI) were calculated to assess the relationship between *NOX5* gene polymorphisms along and in combinations, and the risk of T2D. The PLINK software v.1.9 ( 19 ) was used to evaluate the SNP-phenotype associations. To account for multiple testing, the false discovery rate (FDR) was computed for each SNP-disease relationship (calculations were done using the online FDR calculator at http://www.sdmproject.com/utilities/?show=FDR ). A validation of the observed SNP-disease associations was performed in independent populations whose genotype data are deposed in the T2D Knowledge portal (URL: http://www.type2diabetesgenetics.org ) and the UK Biobank ( http://geneatlas.roslin.ed.ac.uk ). Linear regression analysis was used to assess the impact of *NOX5* polymorphisms on log-normalized biochemical parameters such as HbA1c, FGB, C-peptide, total cholesterol, high- and low-density lipoproteins, triacylglycerol, urea, uric acid, hydrogen peroxide, and total glutathione. The threshold for statistical significance was set at P < 0.05.

## RESULTS ASSOCIATION BETWEEN OF THE NOX5 GENE POLYMORPHISMS AND T2D RISK

As shown in Table 1 , the genotype frequencies of the *NOX5* polymorphisms were in Hardy-Weinberg equilibrium in both cases and controls (P > 0.05). The rs35672233-T allele (OR = 1.63. 95% CI 1.28-2.07. P < 0.0001) and genotype rs35672233-C/T (OR = 1.67. 95% CI 1.29-2.17. P = 0.0002) were found to be associated with an increased risk of type 2 diabetes, whereas other *NOX5* polymorphisms were not. Association analysis stratified by body mass index (BMI) revealed that three SNPs of *NOX5* , namely rs35672233, rs2036343, and rs438866, were associated with the risk of T2D in subjects with BMI ≥ 25 kg/m^2^ regardless sex and age ( Table 1 ). However, only the association between rs35672233 and T2D risk in patients with a BMI ≥ 25 kg/m^2^ remained significant after correction for multiple testing (FDR = 0.005). Meanwhile, none of the *NOX5* polymorphisms was associated with T2D risk in subjects with BMI ≤ 25 kg/m^2^ ( Table 1 ). None of the investigated SNPs was associated with diabetic complications, including retinopathy, nephropathy, neuropathy, angiopathy of the lower extremities, or diabetic foot syndrome (data not shown).

**Table 1:** Genotype and allele frequencies for the studied polymorphisms in T2D patients and healthy controls

*NOX5* SNP ID		n (%)^a^			
	Genotype/allele	Controls (n=1,627)	T2D patients (n=1,579)	adj^P(Q)b^	_adj_OR (95% CI)^c^
**Entire group**					
rs35672233 C>T	C/C C/T	1,515 (93.1) 110 (6.8)	1,409 (89.2) 164 (10.4)	**0.0002 (0.003)**	1.00 **1.67 (1.29-2.17)**
	T/T	2 (0.1)	6 (0.4)		2.63 (0.52-13.28)
rs3743093 A>G	A/A	533 (32.8)	536 (34)	0.79 (0.98)	1.00
	A/G	809 (49.7)	764 (48.4)		0.95 (0.81-1.11)
	G/G	285 (17.5)	279 (17.7)		0.97 (0.78-1.20)
	A/A	1,523(93.6)	1,456 (92.2)		1.00
rs2036343 A>C	C/A	96 (5.9)	120 (7.6)	0.059 (0.17)	1.30 (0.97-1.73)
	C/C	8 (0.5)	3 (0.2)		0.36 (0.09-1.41)
rs311886 C>T	C/C	1,507 (92.6)	1,468 (93)	0.92 (0.98)	1.00
	T C	116 (7.1)	108 (6.8)		0.95 (0.72-1.26)
	T T	4 (0.2)	3 (0.2)		0.83 (0.18-3.91)
	T T	435 (26.7)	446 (28.2)		1.00
rs438866 T>C	T C	858 (52.7)	766 (48.5)	0.089 (0.22)	0.88 (0.75-1.05)
	C C	334 (20.5)	367 (23.2)		1.07 (0.87-1.32)
**BMI < 25 kg/m^2^**					
	C C	279 (92.7)	184 (90.6)		1.00
rs35672233 C>T	C T	22 (7.3)	19 (9.4)	0.35 (0.65)	1.37 (0.71-2.67)
	T T	0	0		-
	A A	102 (33.8)	77 (37.8)		1.00
rs3743093 *A>G*	A/G	152 (50.3)	93 (45.6)	0.61 (0.95)	0.82 (0.55-1.23)
	G/G	48 (15.9)	34 (16.7)		0.95 (0.55-1.64)
	A A	274 (92.3)	189 (95)		1.00
rs2036343 A>C	C A	21 (7.1)	10 (5)	0.19 (0.40)	0.65 (0.30-1.45)
	C C	2 (0.7)	0 (0)		0.00 (0.00-NA)
rs311886 C>T	C C	283 (93.7)	192 (94.1)	1.0 (1.0)	1.00
	T C	18 (6)	11 (5.4)		1.03 (0.47-2.27)
	T T	1 (0.3)	1 (0.5)		1.06 (0.06-17.63)
	T T	83 (27.7)	63 (31)		1.00
rs438866 T>C	T C	151 (50.3)	103 (50.7)	0.55 (0.91)	0.95 (0.62-1.45)
	C C	66 (22)	37 (18.2)		0.75 (0.44-1.28)
**BMI > 25 kg/m^2^**					
	C/C	1,199 (93.2)	1,217 (89)		1.00
rs35672233 C>T	C T	85 (6.6)	144 (10.5)	**0.0006 (0.0045)**	**1.70 (1.28-2.25)**
	T T	2 (0.2)	6 (0.4)		2.41 (0.48-12.18)
	A A	422 (32.5)	457 (33.4)		1.00
rs3743093 *A>G*	A G	643 (49.6)	668 (48.8)	0.90 (0.98)	0.96 (0.81-1.14)
	G G	232 (17.9)	245 (17.9)		0.97 (0.77-1.21)
	A A	1,198 (93.9)	1,248 (91.8)		1.00
rs2036343 A>C	C A	72 (5.6)	108 (8)	**0.021** (0.07)	**1.49 (1.09-2.04)**
	C C	6 (0.5)	3 (0.2)		0.46 (0.11-1.86)
rs311886 C>T	C C	1,194 (92.3)	1,241 (92.8)	0.81 (0.98)	1.00
	T C	96 (7.4)	94 (7)		0.95 (0.70-1.28)
	T T	3 (0.2)	2 (0.2)		0.61 (0.10-3.71)
rs438866 T>C	T T	342 (26.5)	377 (27.8)	**0.019** (0.07)	1.00
	T C	687 (53.3)	653 (48.2)		0.87 (0.72-1.04)
	C/C	261 (20.2)	325 (24)		1.14 (0.91-1.42)

^a^ Absolute number and percentage of individuals/chromosomes with particular genotype/allele.

^b^ Significance value of association (P) adjusted for age and gender with one degree of freedom (Significance value of association, Q adjusted for multiple testing). ^c^ Odds ratio with 95% confidence intervals adjusted for age and gender with one degree of freedom (codominant model). Bold is statistically significant P-and Q-values.

### Replication for the observed SNP-disease associations in independent cohorts

Supplementary Table 2 shows the results of the replication analysis for associations between *NOX5* polymorphisms and T2D risk in independent populations of the UK Biobank and other cohorts. As shown in Supplementary Table 2 , the association of the rs35672233 polymorphism with T2D susceptibility, established in the present study, was not validated in any of the investigated populations. However, polymorphisms rs3743093, rs311886, and rs438866 of *NOX5* showed nominal associations with T2D susceptibility in cohorts, such as Go Darts Illumina Infinium GWAS (P = 0.048), UK Biobank 2 (P = 0.006), and Go Darts Illumina Infinium GWAS (P = 0.039), respectively.

### The joint effects of the NOX5 polymorphisms on the risk of T2D

We investigated whether combinations of *NOX5* genotypes (diplotypes) jointly contributed to T2D susceptibility. As shown in Table 2 , seven *NOX5* diplotypes were significantly (Q < 0.05) associated with the risk of T2D (associations between all diplotypes and T2D are shown in Supplementary Table 3 ). In particular, five diplotypes, rs35672233-C/T×rs3743093-A/G, rs35672233-C/T×rs2036343-A/A, rs35672233-C/T×rs311886-C/C, rs2036343-A/C×rs3743093-A/G, and rs2036343-A/C×rs438866-C/C, were associated with an increased T2D risk, whereas two diplotypes, rs35672233-C/C×rs2036343-A/A and rs35672233-C/C×rs438866-T/C, were associated with a decreased risk. The estimated haplotype frequencies of *NOX5* and their associations with T2D risk are shown in Table 3 . The *H3* haplotype (rs35672233T-rs3743093G-rs2036343A-rs311886C-rs438866C), comprising the minor allele rs35672233-T, was significantly associated with an increased risk of type 2 diabetes (OR = 1.65. 95% CI 1.27-2.13. P = 0.0001. FDR = 0.002) in the entire group and in a subgroup of patients with BMI ≥ 25 kg/m^2^ (OR = 1.67. 95% CI 1.27-2.20. P = 0.0003. FDR = 0.003). None of the other *NOX5* haplotypes were associated with T2D susceptibility. SNP rs438866 was in positive linkage disequilibrium (D` ≥ 0.8, P < 2×10^-6^) with rs35672233, rs3743093, rs2036343, and rs311886, and in negative linkage disequilibrium with rs35672233 and rs2036343 polymorphisms ( Supplementary Table 4 ).

**Table 2:** Statistically significant associations of *NOX5* genotype combinations withT2D risk

Genotype combination	T2D patients		Controls		P-value	Q-value	OR (95% CI)
	n^a^	%^b^	_n_a	%^b^			
rs35672233-C/Txrs3743093-A/G	106	6.8	67	4.2	**0.002**	**0.02**	**1.64 (1.19-2.24)**
rs35672233-C/Cxrs2036343-A/A	1,271	81.9	1,353	87.0	**0.000097**	**0.0068**	**0.68 (0.56-0.82)**
rs35672233-C/Txrs2036343-A/A	153	9.9	99	6.4	**0.00035**	**0.007**	**1.61 (1.24-2.09)**
rs35672233-C/Txrs311886-C/C	153	10.0	103	6.5	**0.0005**	**0.007**	**1.58 (1.22-2.05)**
rs35672233-C/Cxrs438866-T/C	659	42.5	764	48.7	**0.00047**	**0.007**	**0.78 (0.68-0.90)**
rs2036343-A/Cxrs3743093-A/G	53	3.4	26	1.7	**0.0019**	**0.02**	**2.09 (1.30-3.35)**
rs2036343-A/Cxrs438866-C/C	56	3.6	25	1.6	**0.0004**	**0.007**	**2.31 (1.43-3.72)**

^a^ Absolute number of individuals with particular genotype combination. ^b^ Percentage of individuals with particular genotype combination.

T2D, type 2 diabetes; OR, odds ratio; CI, confidence interval. Bold is statistically significant P- and Q-values.

Minor alleles are underlined.

**Table 3:** Estimated haplotype frequencies in T2D patients and controls

*H* \sNPs *\*	rs35672233	3 0 7 3 _r_s	rs2036343	rs311886	_6_6 8 8 _4_3 _r_s	Controls	T2D Patients	OR (95% CI)b	*P (Q)^c^*
**Entire group Global haplotype association P-value: 0.0018**									
*H1*	C	A	A	C	T	0.5199	0.517	1.00	---
*H2*	C	G	A	C	C	0.3402	0.3213	0.95 (0.85-1.06)	0.34 (0.74)
*H3*	T	G	A	C	C	0.0346	0.0556	**1.65 (1.27-2.13)**	**0.0001 (0.0018)**
*H4*	C	A	C	C	C	0.0357	0.0396	1.09 (0.83-1.43)	0.54 (0.74)
*H5*	C	G	A	T	C	0.0383	0.0348	0.94 (0.71-1.24)	0.65 (0.78)
*H6*	C	A	A	C	C	0.0208	0.024	1.27 (0.89-1.82)	0.19 (0.60)
*Rare^1^*	*	*	*	*	*	0.0083	0.0062	0.80 (0.43 - 1.49)	0.49 (0.74)
**BMI<25 kg/m^2^ Global haplotype association P-value: 0.75**									
*H1*	C	A	A	C	T	0.5189	0.5512	1.00	-
*H2*	C	G	A	C	C	0.3312	0.3068	0.87 (0.65 - 1.16)	0.35 (0.74)
*H3*	T	G	A	C	C	0.0352	0.0457	1.29 (0.65 - 2.55)	0.47 (0.74)
*H4*	C	A	C	C	C	0.0371	0.0257	0.59 (0.27 - 1.28)	0.18 (0.60)
*H5*	C	G	A	T	C	0.0316	0.0319	0.98 (0.48 - 2.00)	0.96 (0.96)
*H6*	C	A	A	C	C	0.0334	0.0285	0.85 (0.39 - 1.88)	0.70 (0.78)
*Rare^1^*	*	*	*	*	*	0.0039	0.0090	0.91 (0.26 - 3.12)	0.88 (0.93)
**BMI>25 kg/m^2^ Global haplotype association P-value: 0.0013**									
*H1*	C	A	A	C	T	0.5202	0.5119	1.00	-
*H2*	C	G	A	C	C	0.3426	0.3238	0.96 (0.85-1.09)	0.54 (0.74)
*H3*	T	G	A	C	C	0.0344	0.0569	**1.67 (1.27-2.20)**	**0.0003 (0.0027)**
*H4*	C	A	C	C	C	0.0353	0.0416	1.21 (0.91-1.62)	0.20 (0.60)
*H5*	C	G	A	T	C	0.0396	0.0351	0.92 (0.69-1.24)	0.60 (0.77)
*H6*	C	A	A	C	C	0.0179	0.0233	1.43 (0.95-2.14)	0.085 (0.51)
*Rare^a^*	*	*	*	*	*	0.0090	0.0055	0.77 (0.40-1.50)	0.44 (0.74)

^a^ Rare haplotypes with frequency < 0.01 are not shown. ^b^0dds ratio with 95% confidence intervals adjusted for age and gender.

^c^ Significance value of association adjusted for age and gender (P) and significance value corrected for multiple testing (Q|. T2D, type 2 diabetes; OR, odds ratio; CI, confidence interval. Bold is statistically significant *P-* and Q-values. Minor alleles are underlined.

### The joint effects of NOX5 polymorphisms and risk factors on T2D susceptibility

Since type 2 diabetes mellitus is a multifactorial disorder, it would be reasonable to investigate whether known environmental risk factors of type 2 diabetes interact with *NOX5* polymorphisms to determine disease susceptibility. The results of the gene-environment interaction analysis are presented in Table 4 . The rs35672233-C/T genotype showed synergistic effects on disease risk with three risk factors: chronic psycho-emotional stress (OR = 1.95, 95% CI 1.22-3.12, P = 0.015), sedentary lifestyle (OR = 2.27, 95% CI 1.50-3.43, P = 0.0002), and high-calorie diet (OR = 1.81, 95% CI 1.15-2.86, P = 0.019). However, only the association between the rs35672233-C/T genotype and sedentary lifestyle and T2D risk remained significant after multiple testing corrections (FDR = 0.006).

**Table 4:** Interaction between *NOX5* gene polymorphisms and environmental factors

		No risk factor (f-)				Presence of risk factor (f+)		
Genotypes	Controls, n (%)^a^	T2D patients, n (%)^a^	OR (95% CI)^b^	P (Q)	Controls, n (%)^a^	T2D patients, n (%)^a^	OR (95% CI)^b^	P (Q)
**1**	**2**	**3**	**4**	5	6	**7**	**8**	**9**
**Chronic psychological stress and rs35672233 *NOX5***								
C/C	987(92.8)	526 (89.8)	1.00		491 (93.7)	604 (88.3)	1.00	**0.015** (0.15)
C/T	76 (7.2)	58 (9.9)	1.57 (1.03-2.40)	0.054	31 (5.9)	77 (11.3)	**1.95 (1.22-3.12)**	
T/T	0 (0)	2 (0.3)	NA (0.00-NA)	(0.31)	2 (0.4)	3 (0.4)	0.63 (0.09-4.30)	
**Sedentary lifestyle and rs35672233 *NOX5***								
C/C	924(93)	366 (91.7)	1.00		554 (93.3)	1035 (88.4)	1.00	**0.0002 (0.006)**
C/T	69 (7)	33 (8.3)	1.16 (0.70-1.92)	0.56	38 (6.4)	130 (11.1)	**2.27 (1.50-3.43)**	
T/T	-	-	-	(0.80)	- 2 (0.3)	6 (0.5)	0.84 (0.16-4.45)	
**High calorie diet and rs35672233 *NOX5***								
C/C	983(92.8)	240 (89.2)	1.00		495 (93.8)	1061(89)	1.00	0.019
C/T	74 (7)	29 (10.8)	1.62 (1.03-2.55)	0.081	33 (6.2)	125 (10.5)	**1.81 (1.15-2.86)**	(0.21)
T/T	2 (0.2)	0 (0)	0.00 (0.00-NA)	(0.34)	0 (0)	6 (0.5)	NA (0.00-NA)	
**High calorie diet and rs438866 *NOX5***								
T/T	282 (26.6)	85 (31.9)	1.00		143 (27)	323 (27.3)	1.00	**0.02** (0.21)
T/C	555 (52.3)	136 (51.1)	0.81 (0.59-1.10)	0.12	283 (53.5)	572 (48.4)	0.96 (0.72-1.27)	
C/C	224 (21.1)	45 (16.9)	0.66 (0.44-0.99)	(0.41)	103 (19.5)	288 (24.3)	**1.46 (1.03-2.07)**	

^a^ Absolute number and percentage of individuals/chromosomes with particular genotype/allele.

^b^ Odds ***ratio*** with 95% confidence intervals adjusted for age and BMI with one degree of freedom. Bold is statistically significant P-values/ORs (95%CI).

### Relationship between NOX5 polymorphisms and biochemical parameters in diabetic patients

It would be interesting to investigate the relationship between *NOX5* haplotypes and redox (ROS and glutathione) and other biochemical parameters in the plasma of patients with type 2 diabetes. The haplotype rs35672233C-rs3743093A-rs2036343C-rs311886C-rs438866C was associated with increased levels of total glutathione (Diff = 1.11 µmol/L, 95% CI = 0.19-2.03, P = 0.02), uric acid (Diff = 6.99 µmol/L, 95% CI = 4.96-9.02, P < 0.0001), and decreased levels of two-hour postprandial glucose (Diff = -1.75 mmol/L, 95% CI = -3.0 - -0.51, P = 0.006) in the plasma of type 2 diabetics. No significant associations of *NOX5* haplotypes were observed with other biochemical parameters in the plasma, such as FGB, HbA1c, C-peptide, total cholesterol, LDL, HDL, triglycerides, and urea (data not shown).

## DISCUSSION

Nicotinamide adenine dinucleotide phosphate oxidase is a membrane-bound multi-subunit enzyme complex that utilizes NADPH to produce superoxide anions and other ROS ( 20 ). In contrast to the neutrophilic type of NADPH oxidase, its vascular type, including NOX5, produces radicals mainly intracellularly and for a longer time than the neutrophilic isoforms ( 21 ). NOX5 is a calcium-dependent NADPH oxidase involved in the endothelial ROS production, proliferation, cell growth, angiogenesis, apoptosis, and endothelial response to thrombin ( 22 , 23 ). Our previous studies have established that polymorphisms in various subunits of NADPH oxidase are associated with susceptibility to type 2 diabetes and disease outcomes ( 9 - 10 , 12 , 24 ). The present study is the first to show that genetic variations in *NOX5* contribute to the development of type 2 diabetes. Genotype rs35672233-C/T and haplotype rs35672233T-rs3743093G-rs2036343A-rs311886C-rs438866C of NOX5 were found to be associated with an increased risk of T2D. However, the relationship between the rs35672233 polymorphism and disease risk occurred only in subjects with BMI greater than 25 kg/m^2^. In addition, seven *NOX5* diplotypes showed joint effects on disease risk. We did not find any association between *NOX5* polymorphisms and common T2D complications. Furthermore, environmental risk factors, such as chronic psycho-emotional stress, sedentary lifestyle, and a high-calorie diet, showed synergistic effects with the rs35672233 genotype on T2D susceptibility. A haplotype comprising the allele rs35672233-C protective against T2D was correlated with increased levels of total glutathione and uric acid and decreased levels of two-hour postprandial glucose in the plasma of patients with diabetes.

Thus, among all the studied SNPs, the rs35672233 polymorphism is of interest, namely the carriage of the T allele, both as part of the *NOX5* genotype and haplotype, which was associated with an increased risk of T2D. There have been no functional studies on this polymorphism in the literature. SNP rs35672233 is a non-coding transcript intron variant located in genomic region spanning *NOX5* and *SPESP1* genes. Functional annotation of SNP rs35672233 of *NOX5* using the GTEx portal (https://www.gtexportal.org/home/) and eQTLGen Consortium (https://www.eqtlgen.org/phase1.html) showed that this polymorphism has no significant cis-and trans-eQTLs or, in other words, does not affect expression levels of any gene. Nevertheless, according to VannoPortal (http://www.mulinlab.org/vportal/index.html), rs35672233 is a functional polymorphism that may affect the pancreatic expression levels of *ITGA11* (integrin subunit alpha 11 ) located in the genomic region 15q23 belonging to *NOX5* . According to the Haploreg tool v.4.2 (https://pubs.broadinstitute.org/mammals/haploreg/haploreg.php), SNP rs35672233 may be a part of the enhancer affecting gene expression in the pancreas through the histone mark H3K4Me1. H3K4me1 is a chromatin signature enriched in active and primed enhancers ( 25 ). According to rSNPBase 3.1 database (http://rsnp3.psych.ac.cn/), rs35672233 is an important region associated with the binding of transcription factors such as ZNF263 (zinc finger protein 263), MAX (myc-associated factor X), and TCF12 (transcription factor 12). The last two transcription factors are of interest because they represent Myc, WNT and MAPK signaling pathways known to be involved in the regulation of beta cell physiology and glucose homeostasis, as well as contributing to the pathogenesis of type 2 diabetes and its complications ( 26 , 27 ). Although we did not find an association between the rs35672233 polymorphism and ROS levels, the disease-protective allele C was correlated with increased levels of antioxidants (glutathione and uric acid) as well as decreased levels of two-hour postprandial plasma glucose. These findings indirectly indicate a pro-oxidant effect of the rs35672233-T allele, affecting the expression and/or activity of *NOX5* . However, before drawing definitive conclusions, experimental studies are necessary to functionally assess the effects of this polymorphism.

Experimental studies showed that the negative diabetogenic effects of *Nox5* overexpression have been associated with upregulation of ROS-sensitive factors, oxidative injury, inflammation, and sclerosis of the kidneys in a mouse model of diabetic nephropathy, whereas silencing *Nox5* was found to attenuate hyperglycemia and ROS production ( 13 ). Although NOX enzymes are considered crucial controllers of physiological insulin secretion, they can have negative effects if they are consistently overproduced ( 28 , 29 ). A persistent increase in ROS has been associated with decreased insulin release ( 30 ). Notably, a gene expression study (GEO profiles, ID:71220505) revealed elevated *NOX5* mRNA levels in the islets of patients with type 2 diabetes compared to the islets of non-diabetic subjects, supporting the role of oxidative stress in beta-cell dysfunction in diabetes ( 31 , 32 ). An experimental study in mice demonstrated that beta-cell-specific expression of Nox5 might be an important factor aggravating the high-fat diet-induced impairment of islet insulin secretion and β-cell failure in type 2 diabetes ( 33 ).

Our study has some limitations. Only a limited number of *NOX5* polymorphisms were investigated in the present study. Further studies with a wider spectrum of *NOX5* polymorphisms, including those that showed associations with disease risk in some independent populations ( Supplementary Table 2 ), are required to substantiate the role of this gene in T2D susceptibility. SNP-phenotype associations observed in the subgroup analysis focusing on biochemical parameters and gene-environment interactions were observed in small samples, decreasing the statistical power and reliability of the findings. Finally, the observed SNP-disease associations should be interpreted with caution, since no functional studies of the investigated polymorphisms have been performed.

In conclusion, the present study is the first to establish associations between polymorphisms in *NOX5* and the risk of type 2 diabetes mellitus, and provides a new line of evidence for the crucial role of oxidative stress-related genes in disease susceptibility. Associations between *NOX5* polymorphisms and disease risk have been found in individuals with diabetes risk factors, such as an increased body mass index, sedentary lifestyle, chronic psychological stress, and a high-calorie diet. These associations can be explained by the fact that the influence of these factors is associated with enhanced production of ROS and oxidative stress ( 34 - 38 ), and thus potentiates the pro-oxidant effects of NOX5, increasing the risk of type 2 diabetes. However, this assumption requires experimental confirmation. Nevertheless, the impact of *NOX5* polymorphisms on disease risk is modulated by well-known environmental risk factors, which justifies the crucial role of lifestyle interventions in type 2 diabetes prevention programs. Although the rs35672233 polymorphism has not been replicated in independent cohorts as a susceptibility marker for diabetes, three other SNPs, namely rs3743093, rs311886, and rs438866, showed nominal associations with disease risk in at least one of the independent populations, suggesting a potential role for the *NOX5* gene in the development of type 2 diabetes. Further studies will shed light on the molecular mechanisms underlying the relationship between the gene and T2D pathogenesis and show whether *NOX5* is a target gene for the pharmacological inhibition of oxidative stress in diabetes and its complications.

## SUPPLEMENTARY

**Supplement Table 1:** Primer sequences

Primer sequence 5'-*3"			
SNP ID	**Forward**	**Reverse**	**Extended**
rs35672233 C>T	ACGTTGGATGTTAATGCCCTTGCCTACCTC	ACGTTGGATGGAACTGAGCATAGTTCCGGC	ACTGCCCTCCCAAGC
rs3743093 A>G	ACGTTGGATGGTCACCTGTCACCACTTTAG	ACGTTGGATGGAGAGCTGAGGAACATCTTC	gAGAGCACTGGCATTG
rs2036343 A>C	ACGTTGGATGCCTTAACTGGCCATAACTTG	ACGTTGGATGGTTCAGGAATAATATGTAGG	TGGCTTATTTCCTCAAAAG
rs311886 C>T	ACGTTGGATGCCAAAGGGTGGCTATAAGTC	ACGTTGGATGCTCATGTCCCTGAGCTGAAG	agacCTGCCCCCCCAAAGACATTCAC
rs438866 T>C	ACGTTGGATGGCATCAACTGGTTACCACTG	ACGTTGGATGTACCACAGCAGTGAATCTCC	ttttTCCATTAATGACACCCA

**Supplement Table 2:** Replication of associations and identification of interpopulation differences in the associations of *N0X5* SNPs with the risk of T2D

Gene SNP	Minor allele	'Central Russia	^2^UK Biobankl	^3^UK Biobank2	'EXTEND GWAS	"Go Darts lllumina Infinium GWAS	"Go Darts exome chip analysis	4FUSI0N GWAS	'Diabetic Cohort Singapore Prospective Study GWAS	'AMP T2D-GENES exome sequence analysis	'CAMP GWAS	'BioMe AMPT2D GWAS	'METSIM GWAS
1	2	3	4	5	6	7	8	9	10	11	12	13	14
*N0X5*	Τ	**0.0002**	0.19	0.53	0.0867	0.204	-	-	-	-	-	0.436	0.402
C>T													
rs35672233													
*N0X5*	G	0.79	0.77	0.89	0.569	**0.0481**	0.677	0.418	0.552	0.717	0.732	0.627	0.333
A>G													
rs3743093													
*N0X5*	Τ	0.92	0.19	**0.0059**	0.671	0.0862	-	-	-	-	-	-	-
C>T													
rs311886													
*N0X5*	C	0.089	0.97	0.89	0.416	**0.0392**	-	-	0.782	-	0.321	0.870	0.174
T>C													
rs438866													

^1^Data on the significance levels of associations of the studied SNPs with the risk of T2D in the population of Central Russia adjusted for sex, age, and BMI.

^1^Data on the significance levels of associations of the studied SNPs with the risk of T2D in the population of UK Biobank (type 2 diabetes mellitus] adjusted for sex, age, and BMI (Gene Atlas http://geneatlas.roslln.ed.ac.uk ).

^3^Data on the significance levels of associations of the studied SNPs with the risk of T2D in the population of UK Biobank (ποπ-insulin-dependent diabetes mellitus) adjusted for sex, age, and BMI (Gene Atlas http://geneatlas.roslin.ed.ac.uk ).

^4^Data on the significance levels of associations of the studied SNPs with the risk of T2D in the population of T2D knowledge Portal adjusted for sex, age, and BMI ( http://www.type2diabetesgenetics.org ).

**Supplement Table 3:** Associations of *NOX5* genotype combinations withT2D risk

Genotype combination	T2D patients		Controls		P-value	Q-value	OR 95% CI
	n^1^	%^2^	n^1^	%^2^			
rs35672233-C/Cxrs3743093-A/A	534	34.1	521	33.0	0.54	0.77	1.05 (0.90-1.21)
rs35672233-C/Cx rs3743093-A/G	650	41.5	716	45.4	0.03	0.17	0.85 (0.74-0.98)
rs35672233-C/Cx rs3743093-G/G	214	13.7	232	14.7	0.40	0.73	0.92 (0.75-1.12)
rs35672233-C/Tx rs3743093-A/G	106	6.8	67	4.2	**0.002**	**0.02**	**1.64 (1.19-2.24)**
rs35672233-C/Tx rs3743093-G/G	57	3.6	39	2.5	0.06	0.30	1.49 (0.98-2.25)
rs35672233-T/Tx rs3743093-G/G	6	0.4	2	0.1	0.28	0.72	2.62 (0.61-11.30)
rs35672233-C/Cxrs2036343-A/A	1271	81.9	1353	87.0	**0.000097**	**0.0068**	**0.68 (0.56-0.82)**
rs35672233-C/Cx rs2036343-A/C	109	7.0	89	5.7	0.14	0.49	1.25 (0.93-1.66)
rs35672233-C/Cx rs2036343-C/C	3	0.2	7	0.5	0.34	0.73	0.47 (0.13-1.66)
rs35672233-C/Tx rs2036343-A/A	153	9.9	99	6.4	**0.00035**	**0.007**	**1.61 (1.24-2.09)**
rs35672233-C/Tx rs2036343-A/C	9	0.6	4	0.3	0.26	0.72	2.12 (0.69-6.53)
rs35672233-C/Tx rs2036343-C/C	0	0.0	1	0.1	1.00	1.00	0.33 (0.01-8.21)
rs35672233-T/Tx rs2036343-A/A	6	0.4	2	0.1	0.29	0.72	2.61 (0.61-11.26)
rs35672233-C/Cxrs311886-C/C	1268	82.7	1351	85.8	**0.02**	**0.13**	**0.79 (0.65-0.96)**
rs35672233-C/Cx rs311886-C/T	97	6.3	111	7.1	0.42	0.73	0.89 (0.67-1.18)
rs35672233-C/Cx rs311886-T/T	3	0.2	4	0.3	0.97	1.00	0.80 (0.20-3.23)
rs35672233-C/Tx rs311886-C/C	153	10.0	103	6.5	**0.0005**	**0.007**	**1.58 (1.22-2.05)**
rs35672233-C/Tx rs311886-C/T	7	0.5	3	0.2	0.32	0.73	2.20 (0.62-7.86)
rs35672233-T/Tx rs311886-C/C	6	0.4	2	0.1	0.27	0.72	2.67 (0.62-11.52)
rs35672233-C/Cxrs438866-T/T	440	28.4	423	27.0	0.39	0.73	1.07 (0.92-1.25)
rs35672233-C/Cxrs438866-T/C	659	42.5	764	48.7	**0.00047**	**0.007**	**0.78 (0.68-0.90)**
rs35672233-C/Cxrs438866-C/C	287	18.5	275	17.5	0.48	0.75	1.07 (0.89-1.28)
rs35672233-C/Txrs438866-T/T	0	0.0	1	0.1	1.0	1.00	0.34 (0.01-8.27)
rs35672233-C/Txrs438866-T/C	92	5.9	60	3.8	0.01	0.08	1.58 (1.14-2.21)
rs35672233-C/Txrs438866-C/C	67	4.3	43	2.7	0.02	0.12	1.60 (1.08-2.36)
rs35672233-T/Txrs438866-C/C	6	0.4	2	0.1	0.28	0.72	2.64 (0.61-11.35)
rs3743093-A/Axrs2036343-A/A	459	29.5	439	28.1	0.38	0.73	1.07 (0.92-1.25)
rs3743093-A/Axrs2036343-A/C	64	4.1	65	4.2	0.95	1.00	0.99 (0.70-1.41)
rs3743093-A/Axrs2036343-C/C	2	0.1	6	0.4	0.29	0.72	0.39 (0.09-1.66)
rs3743093-A/Gxrs2036343-A/A	701	45.1	750	48.0	0.10	0.37	0.89 (0.77-1.02)
rs3743093-A/Gxrs2036343-A/C	53	3.4	26	1.7	**0.0019**	**0.02**	**2.09 (1.30-3.35)**
rs3743093-A/Gxrs2036343-C/C	1	0.1	0	0.0	1.0	1.00	3.02 (0.12-74.13)
rs3743093-G//Gxrs2036343-A/A	275	17.7	276	17.6	0.98	1.00	1.00 (0.83-1.20)
rs3743093-G/Gxrs2036343-A/C	1	0.1	1	0.1	0.48	0.74	1.01 (0.10-9.67)
rs3743093-G//Gxrs2036343-C/C	0	0.0	1	0.1	1.00	1.00	0.33 (0.01-8.23)
rs3743093-A/Ax rs311886-C/C	522	33.9	522	32.8	0.53	0.76	1.05 (0.90-1.22)
rs3743093-A/Gx rs311886-C/C	682	44.3	721	45.3	0.55	0.76	0.96 (0.83-1.10)
rs3743093-A/Gx rs311886-C/T	60	3.9	71	4.5	0.43	0.73	0.87 (0.61-1.23)
rs3743093-A/Gx rs311886-T/T	1	0.1	0	0.0	0.99	1.00	3.10 (0.13-76.14)
rs3743093-G/Gx rs311886-C/C	228	14.8	229	14.4	0.75	0.90	1.03 (0.85-1.26)
rs3743093-G/Gx rs311886-C/T	45	2.9	43	2.7	0.71	0.87	1.08 (0.71-1.65)
rs3743093-G/Gx rs311886-T/T	2	0.1	4	0.3	0.71	0.87	0.57 (0.12-2.69)
rs3743093-A/Ax rs438866-T/T	427	27.4	407	25.7	0.27	0.72	1.09 (0.93-1.28)
rs3743093-A/Ax rs438866-T/C	96	6.2	110	6.9	0.38	0.73	0.88 (0.66-1.17)
rs3743093-A/Ax rs438866-C/C	7	0.4	6	0.4	0.97	1.00	1.18 (0.41-3.37)
rs3743093-A/Gx rs438866-T/T	13	0.8	16	1.0	0.61	0.81	0.83 (0.40-1.72)
rs3743093-A/Gx rs438866-T/C	653	41.9	715	45.1	0.07	0.33	0.88 (0.76-1.01)
rs3743093-A/Gx rs438866-C/C	86	5.5	55	3.5	0.01	0.08	1.63 (1.15-2.30)
rs3743093-G/Gx rs438866-T/C	6	0.4	10	0.6	0.47	0.74	0.63 (0.24-1.68)
rs3743093-G/Gx rs438866-C/C	269	17.3	266	16.8	0.71	0.87	1.04 (0.86-1.25)
rs2036343-A/Ax rs311886-C/C	1305	85.7	1347	86.4	0.57	0.77	0.94 (0.77-1.16)
rs2036343-A/Ax rs311886-C/T	97	6.4	110	7.1	0.45	0.74	0.90 (0.68-1.19)
rs2036343-A/Ax rs311886-T/T	3	0.2	4	0.3	0.98	1.00	0.80 (0.20-3.22)
rs2036343-A/Cx rs311886-C/C	110	7.2	89	5.7	0.09	0.35	1.29 (0.96-1.72)
rs2036343-A/Cx rs311886-C/T	5	0.3	2	0.1	0.43	0.73	2.26 (0.51-10.08)
rs2036343-C/Cx rs311886-C/C	3	0.2	7	0.4	0.36	0.73	0.48 (0.13-1.70)
rs2036343-A/Axrs438866-T/T	432	28.1	422	27.1	0.57	0.76	1.05 (0.89-1.23)
rs2036343-A/Ax rs438866-T/C	694	45.1	748	48.1	0.09	0.35	0.89 (0.77-1.02)
rs2036343-A/Ax rs438866-C/C	296	19.2	286	18.4	0.56	0.76	1.06 (0.88-1.26)
rs2036343-A/Cx rs438866-T/T	0	0.0	1	0.1	1.0	1.00	0.34 (0.01-8.26)
rs2036343-A/Cxrs438866-T/£	59	3.8	66	4.2	0.56	0.76	0.90 (0.63-1.29)
rs2036343-A/Cx rs438866-C/C	56	3.6	25	1.6	**0.0004**	**0.007**	**2.31 (1.43-3.72)**
rs2036343-C/Cxrs438866-C/C	3	0.2	7	0.5	0.35	0.73	0.47 (0.13-1.67)
rs311886-C/Cxrs438866-T/T	431	28.2	424	26.8	0.39	0.73	1.07 (0.92-1.25)
rs311886-C/Cxrs438866-T/C	687	45.0	765	48.4	0.06	0.30	0.87 (0.76-1.00)
rs311886-C/Cxrs438866-C/C	303	19.8	275	17.4	0.08	0.35	1.17 (0.98-1.41)
rs311886-C/Txrs438866-T/C	52	3.4	69	4.4	0.17	0.56	0.77 (0.53-1.11)
rs311886-C/Txrs438866-C/C	52	3.4	44	2.8	0.32	0.73	1.23 (0.82-1.85)
rs311886-T/Txrs438866-T/C	1	0.1	0	0.0	0.99	1.00	3.11 (0.13-76.31)
rs311886-T/Txrs438866-C/C	2	0.1	4	0.3	0.71	0.87	0.57 (0.12-2.70)

^1^Absolute number of individuals with particular genotype combination.

^2^Percentage of individuals with particular genotype combination.

T2D, type 2 diabetes; OR, odds ratio; CI, confidence interval. Bold is statistically significant P- and Q-values. Minor alleles are underlined.

**Supplement Table 4:** Linkage disequilibrium between *NOX5* SNPs in the Russian population

SNP ID	rs35672233	rs3743093	rs2036343	rs311886	rs438866
rs35672233	-	0,0262	-0,0009	-0,0016	0,0236
	-	0,9982	0,5059	0,9576	0,9888
rs3743093	-	-	-0,0143	0,0212	0,2146
	-	-	0 9092	0,9878	0,9649
rs2036343	-	-	-	-0,0013	0,0193
	-	-	-	0,9484	0,9846
rs311886	-	-	-	-	0,0193
	-	-	-	-	0,9846

Upper cells show D-values (gray fill); lower cells show £7-values (P<2.0x10^16^).
